# Greenness and excess deaths from heat in 323 Latin American cities: Do associations vary according to climate zone or green space configuration?

**DOI:** 10.1016/j.envint.2023.108230

**Published:** 2023-10

**Authors:** Leah H Schinasi, Maryia Bakhtsiyarava, Brisa N. Sanchez, Josiah L. Kephart, Yang Ju, Sarav Arunachalam, Nelson Gouveia, Waleska Teixeira Caiaffa, Marie S. O'Neill, Iryna Dronova, Ana V. Diez Roux, Daniel A. Rodriguez

**Affiliations:** a Department of Environmental and Occupational Health, Drexel Dornsife School of Public Health, Philadelphia, USA; b Urban Health Collaborative, Drexel Dornsife School of Public Health, Philadelphia, USA; c Institute of Transportation Studies, University of California, Berkeley, CA, USA; d Department of Epidemiology and Biostatistics, Drexel Dornsife School of Public Health, Philadelphia, USA; e School of Architecture and Urban Planning, Nanjing University, Nanjing, China; f Institute for the Environment, University of North Carolina at Chapel Hill, Chapel Hill, USA; g Department of Preventive Medicine, University of Sao Paulo Medical School, Sao Paulo, Brazil; h Observatory for Urban Health in Belo Horizonte, School of Medicine, Federal University of Minas Gerais, Belo Horizonte, Brazil; i Departments of Epidemiology and Environmental Health Sciences, University of Michigan School of Public Health, Ann Arbor, USA; j Department of Environmental Science, Policy & Management, University of California, Berkeley, USA; k Department of Landscape Architecture & Environmental Planning, University of California, Berkeley, USA; l Department of City and Regional Planning and Institute of Transportation Studies, University of California, Berkeley, USA

**Keywords:** Green space, Vegetation, NDVI, Urban heat, Ambient temperature, Climate change, Mortality, Climate zone

## Abstract

Green vegetation may protect against heat-related death by improving thermal comfort. Few studies have investigated associations of green vegetation with heat-related mortality in Latin America or whether associations are modified by the spatial configuration of green vegetation. We used data from 323 Latin American cities and meta-regression models to estimate associations between city-level greenness, quantified using population-weighted normalized difference vegetation index values and modeled as three-level categorical terms, and excess deaths from heat (heat excess death fractions [heat EDFs]). Models were adjusted for city-level fine particulate matter concentration (PM_2.5_), social environment, and country group. In addition to estimating overall associations, we derived estimates of association stratified by green space clustering by including an interaction term between a green space clustering measure (dichotomized at the median of the distribution) and the three-level greenness variable. We stratified analyses by climate zone (arid vs. temperate and tropical combined). Among the 79 arid climate zone cities, those with moderate and high greenness levels had modestly lower heat EDFs compared to cities with the lowest greenness, although protective associations were more substantial in cities with moderate versus high greenness levels and confidence intervals (CI) crossed the null (Beta: −0.41, 95% CI: −1.06, 0.25; Beta −0.23, 95% CI: −0.95, 0.49, respectively). In 244 non-arid climate zone cities, associations were approximately null. We did not observe evidence of effect modification by green space clustering. Our results suggest that greenness may offer modest protection against heat-related mortality in arid climate zone Latin American cities.

## Highlights

• Few studies have investigated links between greenness and heat-related mortality in Latin American settings.
• Few studies have investigated if associations between greenness and heat-related death vary according to climate zone, or according to the spatial configuration of green space.
• We observed modest protective associations between greenness and heat-related deaths in Latin American cities located in arid climate zones.

## 1 Introduction

Sometimes touted as a sign of progress and growth, urbanization processes have profound effects on the natural environment and energy balance within cities. This is exemplified by the urban heat island effect, which refers to the observation that urban areas are hotter than adjacent suburban or rural areas. The urban heat island effect is explained by various characteristics in the urban environment, including low amounts of green space, high amounts of heat-absorbing impervious surfaces including concrete and asphalt, tall buildings contributing to trapping of direct solar radiation, and anthropogenic heat production (Oke, 1982, Kalnay and Cai, 2003). Urbanization processes have also contributed to green space fragmentation (Li et al., 2019), which has implications for energy flow and exchange, and thus, heat intensity within cities (Masoudi and Tan, 2019). Taken together, the environmental impacts of urbanization combined with globally rising temperatures represents a critical human health concern. Indeed, over 50% of the human population now lives in cities (Grimm et al., 2008, UN, Department of Economic and Social A airs, 2018), and there is strong empirical evidence that extreme heat contributes to excess premature death (Gasparrini et al., 2015, Laaidi et al., 2012). There is a critical need to identify and design optimal city-level interventions to reduce heat vulnerability, which is “…the propensity or predisposition to be adversely affected…” (Pörtner et al., 2022).

Increasingly, municipalities are establishing goals to enhance the presence of green spaces in urban environments (O’Neill et al., 2009). These goals are supported by evidence that vegetation may mitigate heat intensity at the neighborhood or city level (Stone et al., 2014). Green spaces reduce surface and air temperatures through evapotranspiration, by providing shade, by reducing the need for mechanical cooling (which contributes to urban heat emissions), by reflecting short-wave solar radiation into the atmosphere (Gunawardena et al., 2017), and/or by affecting air movement (Bowler et al., 2010). A simulation study of three U.S. cities showed that increasing both vegetation and albedo (i.e., solar reflectance of surfaces) (Akbari et al., 2012) would offset projected increases in heat related mortality by 40–99%. (Stone et al., 2014) A study in Glasgow, UK suggested that increasing green space by 20% could eliminate between a third to a half of the city’s urban heat island effect by year 2050 (Emmanuel and Loconsole, 2015). From a public health perspective, urban greening interventions are also attractive because of potential co-benefits: exposure and/or access to green spaces has been associated with lower rates of overall and cause-specific mortality, improved mental health, uptake of physical activity, and greater social cohesion, among other population health and well-being benefits (Markevych et al., 2017).

Epidemiologic research exploring links between green space and excess deaths from heat has suggested that higher amounts of green space are associated with modest decreases in heat-related death or illness (Markevych et al., 2017, Schinasi et al., 2018, Sera et al., 2019, Choi et al., 2022, Choi et al., 2022). Yet, little is known about whether associations between green space and heat-related mortality vary according to the spatial configuration of vegetation. Exposure studies have shown that, holding constant the same amount of vegetation, the cooling potential of green space at localized or regional levels varies according to how it is configured (Zhou et al., 2011, Li et al., 2012). This work suggests that it is not just the presence of vegetated landscape elements, but also its configuration, which impacts urban environmental cooling (Monteiro et al., 2016, Kong et al., 2014). However, in the exposure literature, the direction of the association of green space configuration with land surface temperatures has been inconsistent. For example, some studies have observed greater cooling effects when vegetation is more clustered Monteiro et al., 2016, Kong et al., 2014, while others have found that greater distribution of green space and less clustering was associated with cooler land surface temperatures (Zhou et al., 2011).

The variation in findings across studies may be explained, at least in part, by differences in climate region. Some research has suggested that green space may have a less substantial cooling effect in arid climate areas, potentially because of low soil moisture and limited tree canopy coverage in those areas (Wang et al., 2022, Zhou et al., 2017). Other studies have found that urban vegetation cools more effectively in drier climates (Tayyebi and Jenerette, 2016). Because human thermal comfort is determined by a combination of surface and radiant temperatures, air humidity, and wind speed, relationships of green space configuration and composition with human heat vulnerability may also vary according to the climate region and local landscaping. For example, in hot tropical climate regions, vegetation may increase humidity levels and thus reduce rather than improve thermal comfort (although it can offer localized comfort through shading) (Jendritzky et al., 2012).

Here, we leveraged a database of 323 large Latin American cities to quantify associations between city-level greenness and excess deaths from heat and investigate if green space clustering (i.e., spatial aggregation) modifies associations. We also investigated whether these relationships vary according to climate zone. First, we hypothesized that we would observe protective associations between greenness and excess deaths from heat. Second, we hypothesized that green space clustering would modify the association of greenness with excess deaths from heat. We also hypothesized that associations between green space and excess deaths from heat would vary according to climate zone. The predominant focus of research on heat vulnerability has been in European, Chinese, or North American cities; however, physiologic and/or behavioral heat adaptation may vary from place to place (Thai et al., 2019). Thus, an innovation of our work is that we quantified these associations in many lower- and middle- income countries of Latin America. Given the impact of climate change in lower and middle income countries of the global South, it is imperative to document the health consequences of increasing temperatures, and the potential modifiers of this impact, in these understudied settings.

## 2 Methods

### 2.1 Time series of ambient temperature and mortality

The data, assembled as a part of the *Salud Urbana en América Latina* (SALURBAL) project (Masoudi and Tan, 2019), were initially restricted to 326 cities in Argentina, Brazil, Chile, Mexico, Peru and Central America (Costa Rica, El Salvador, Guatemala, and Panama) with daily mortality information available. Individual-level mortality data were compiled from vital registration systems in each country. We constructed city-specific time series of daily all-cause mortality counts (World Health Organization Global Health Estimate 2015 classification grouping I., II., and III.) (World Health Organization, 2020) linked with mean daily temperatures in periods ranging from 2002 to 2015. SALURBAL defines cities as urban agglomerations with more than 100,000 residents in year 2010 (Grimm et al., 2008). Causes of death were ascertained from mortality records, coded using the International Classification of Diseases 10th revisions. Mean daily temperatures were estimated as averages of the temperature at two-meters above land surface from the European ReAnalysis 5 (ERA5)-Land climate reanalysis (native ∼ 9 km horizontal resolution). ERA5 is the fifth atmospheric reanalysis that the European Center for medium-range weather forecasts (ECMWF) has produced (Muñoz-Sabater et al., 2021, Hersbach et al., 2020). We calculated daily average temperatures using hourly reanalysis estimates of air temperature at 2 m above the land surface. The ERA5-Land data excludes grid cells containing > 50% water. Because 99 of the 326 cities had at least 1 grid cell missing temperature data, we imputed missing temperatures using a random forest regression model that included resampled ERA5 temperature (31 km resolution), elevation, and aspect. Kriging spatial interpolation was used to perform additional modeling of the residuals. The temperature values were spatially weighted to create a city-level average using 2010 estimates of the population distribution from WorldPop (Argentina, Brazil, Chile, Costa Rica, El Salvador, Guatemala, Mexico, https://www.worldpop.org) (Tatem, 2017) or urban footprint (Global Urban Footprint, Panama and Peru, https://www.un-spider.org/node/11424) (Esch et al., 2017). Further details on temperature derivation and imputation are given elsewhere (Kephart et al., 2022). City level data representing total population and population age composition came from Census Bureaus, National Institutes of Statistics or similar sources for each country. More information on this data compilation has been published previously (Grimm et al., 2008).

### 2.2 Green space measures

#### 2.2.1 Greenness

We measured greenness as the population weighted mean of the annual maximum normalized difference vegetation index (NDVI). NDVI was calculated using remotely sensed images of Moderate Resolution Imaging Spectroradiometer (MODIS) product MOD13Q1.006, supplied at 250 m resolution every 16 days (Pekel et al., 2016, Didan, 2015). MODIS products compute vegetation indices from atmospherically corrected bi-directional surface reflectance, masked for water, clouds, heavy aerosols, and cloud shadows. Permanent and seasonal water was further removed from the NDVI dataset using the European Joint Research Council (JRC) Yearly Water Classification History dataset (High-resolution, 2016). First, the annual maximum NDVI value for every 250 m pixel was identified for each year of the study period; we used the maximum value in order to capture peak vegetation growth (Tucker, 1979). Then, a population weighted area mean of annual maximum NDVI values was calculated using 2010 estimates of the population distribution from WorldPop (https://www.worldpop.org) (Tatem, 2017). Finally, to derive a single value for each city, we calculated the average of the yearly population-weighted mean of annual maximum NDVI values.

#### 2.2.2 Spatial configuration of green space

Green space patches, which are defined as contiguous areas of green area, were delineated using the Moore neighborhood rule wherein a target green space pixel and the eight green space pixels surrounding it were merged into a single green patch. Green space was defined as green grass, shrub, forest, and farmland, and excluded buildings, pavement, roads, barren land, and dry vegetation. Water bodies and limited no observation areas in remote sensing images (due to cloud or shadow) were excluded. The green patches were identified using a green space map that was developed using supervised binary classification of single, wall-to-wall, cloud free median composite of 10 m resolution Sentinel-2 Top of Atmosphere (TOA) reflectance images from year 2017 (Ju et al., 2022). Using images from Sentinel-2, rather than MODIS, was preferable due to Sentinel-2′s higher spatial resolution, which allows for improved characterization and identification of individual patches.

Green space configuration metrics were developed using the FRAGSTATS 4.2 program (McGarigal and Marks, 1995), which is a spatial pattern analysis program that quantifies the spatial distribution of green space patches. Numerous green space configuration metrics are available. We first explored correlations between the following configuration metrics and quantified their predictiveness of excess death fractions from heat (heat-EDFs, described in detail below): mean nearest neighbor distance of green space patches (meters), clumpiness (unitless), and patch density (# patches/hectare). These metrics represent green space patch isolation, aggregation, and fragmentation, respectively (detailed definitions are presented in Appendix 1). Our preliminary analyses indicated high levels of correlation between the isolation and aggregation metrics in temperate and tropical climate zone cities (Pearson Correlation Coefficients = 0.78 and 0.86, respectively), and between fragmentation and NDVI in temperate zone cities (Pearson Correlation Coefficient = 0.58, Appendix 2). Also, of all the configuration metrics, aggregation was most predictive of the heat EDFs. Based on these observations, and to simplify translation of our analyses and results, we focused on green space aggregation using the clumpiness metric (McGarigal and Marks, 1995). Clumpiness, to which we refer hereafter as clustering, is computed based on the spatial adjacency matrix of green patches, which shows the frequency with which different pairs of patches appear side by side in space. This variable ranges between −1 and 1; −1 indicates maximum disaggregation, 0 indicates random distribution, and 1 indicates maximum clustering. In an urban landscape, lower values of clumpiness could be interpreted as landscape filled with a large number of small patches of greenery, whereas values close to 1, the maximum value, would indicate an urban landscape filled with a small number of large green patches (Ha et al., 2022). A map showing green space clustering in Teresopolis, Brazil, which has a relatively high clumpiness index (0.84), and in Catamarca, Argentina, which has a relatively low clumpiness index (0.58), is given in Appendix 3.

### 2.3 Covariates

Averages of city-level annual fine particulate matter concentrations (PM_2.5_, µg/m (Li et al., 2019) corresponding to the same years for which mortality data were available from each city, were calculated using Washington University Atmospheric Composition Analysis Group data (version V5.GL.02) (van Donkelaar et al., 2021, Hammer et al., 2020). A time-invariant social environment index was calculated by first z-score standardizing and then computing the sum of the following four variables: % of the population age 25 and older with at least a primary education; % of households with access to a municipal sewage network; % of households with access to piped water; and % of households with more than three people per room (Bilal et al., 2021). The variable representing crowding was reverse coded so that lower values of the index represent greater social deprivation. The variables for the social environment index were derived from census data of each city. Because we were interested in stratifying analyses by climate zone, we identified the climate zone to which each city belonged using the 1986–2010 version of the Köppen climate classification system (Kottek et al., 2006), and assigned each city to the major two-level category to which the majority of a city’s area belonged.

### 2.4 Analysis

Consistent with previous city-level analyses (Sera et al., 2019), we conducted this as a two-step analysis. We describe these stages below.

### 2.5 Stage 1: Estimating city-specific heat EDFs and relative risk

We have presented details on the methods used to derive city-specific EDFs elsewhere (Kephart et al., 2022). Briefly, we used distributed lag nonlinear models (Gasparrini et al., 2010), fitted within a conditional Poisson regression framework (Armstrong et al., 2014), to estimate the lagged associations between mean daily temperature and all-cause mortality for each city. This was accomplished using a crossbasis function that takes as input the mean daily temperature and the lag day when it occurred, up to 21 lag days. Within the crossbasis function, temperature was modeled as a non-linear term using natural cubic spline functions with knots placed at the minimum, maximum, and 10th, 75th, and 90th percentiles of the city-specific distribution of average daily temperatures. The lag function was modeled as a natural cubic B spline with three internal knots, placed at intervals spaced at equivalent distances from one another on the log scale. The models controlled for seasonality by conditioning on strata defined by day of the week, month and year (Appendix 4). We used the model results to derive city-specific cumulative estimates of associations between temperature and mortality, summed across the 21 lag days (Gasparrini et al., 2010). We used the resulting city-specific non-linear estimates of temperature-mortality associations to identify the daily mean temperature within each city at which the mortality risk was lowest (i.e., the optimal temperature from a health perspective) (Gasparrini et al., 2010). Following existing work, and to not base the reference risk off of the minimum or maximum temperatures, we forced the “optimal temperature” to be between the first and ninety-ninth percentiles of the city-specific temperature distributions. Note that the temperature-mortality association may not follow the typical U- or J-shaped curve among cities that are generally very cold or have a very limited temperature range. For some of those cities, the mortality-temperature association curve may be flat or continue to decrease after the 99th temperature percentile.

We obtained two summary measures to summarize the impact of heat on mortality. First, we used methods developed by others to calculate the fraction of deaths that occurred when temperatures were above the optimal temperature (denoted excess death fractions [EDF] from heat) (Gasparrini and Leone, 2014). Note that in cases where the temperature-mortality curve does not follow the typical U or J-shaped curve but instead continues to decrease after the 99th temperature percentile, the EDF values were, counterintuitively, negative. Second, for each city, we also derived a quantitative estimate of the steepness of the slope of the non-linear temperature-mortality curve between the 95th percentile and the 99th percentiles of the city-specific temperature distributions (denoted the relative risk [RR] of heat related mortality for every 1 °C above hot temperatures). We performed this calculation for the 95th to 99th percentile portions of the curve in order to isolate the steepness of the slope at the highest temperatures for each city. To do this, we used the estimated non-linear temperature-mortality association curve, in the log scale, to compare the log RR when temperatures were at the 99th percentile of the city-level distribution to the log RR when temperatures were at the 95th percentile of the distribution. We then divided this value by the difference (in °C) between the 99th and the 95th percentiles of the temperature distribution in each city (Kephart et al., 2022).

### 2.6 Stage 2: Conducting the meta-regression

We excluded one city from the analysis because it was in a polar climate zone. We excluded two additional cities (from non-arid climate zones) with outlier heat-excess death fraction [EDF] values (i.e., heat EDFs > 10), which we identified based on visual examination of scatter plots. This left 323 cities in the final analysis, all of which were located in climates that were tropical, temperate, or arid (Appendix 5).

We used multivariable random effects linear meta-regression models to estimate associations between greenness and the heat EDFs. (Gasparrini et al., 2012) We selected greenness, measured using the NDVI, rather than percent of land area covered by green space (calculated from the green space map described above) as the primary independent variable because based on minimization of Akaike Information Criterion (AIC) statistics in preliminary analyses, NDVI was more predictive of the heat EDFs than percent green. Further, use of NDVI as the primary independent variable allowed direct comparison with previously conducted research on effect modification of associations between ambient temperature and mortality by green space (Sera et al., 2019, Choi et al., 2022). Because, in preliminary analyses, we found evidence of non-linear associations between NDVI and the heat EDFs, we modeled greenness as a three-level categorical term, with categories based on tertiles of the climate-zone (arid vs. tropical/temperate) specific distributions. The climate zone-specific tertiles are presented in Appendix 6. We included the following variables in models to adjust for potential confounding: average PM_2.5_ concentration across all years, social environment index and country. Cities in Central American countries were grouped together; thus, the variable for country had the following categories: Brazil, Chile, Argentina, Mexico, Peru, and Central America. We also adjusted the main meta-regression model for clustering of green space, modeled as a two-level categorical term with categories based on the median of the climate-zone specific distribution (the medians are reported in Table 1). This two-level parameterization was chosen because it represented different amounts of clustering and allowed sufficient number of cities within each category. PM_2.5_ and the social environment index were modeled as continuous terms. We selected these covariates based on *a priori* hypotheses that they might confound associations and based on the development of a Directed Acyclic Graph (DAG, Appendix 7). Although percent built-up area was identified as a potential confounder of associations based on the DAG, we did not include this covariate in our analyses because it was highly correlated with city-level NDVI, especially in the temperate and arid climate zones (Appendix 2). We included the green space clustering measure (coded as a two-level categorical term) as a covariate in all models to ensure that the main model was nested in the interaction term models. We quantified heterogeneity across cities by computing the Cochran Q test statistic.

**Table 1:** Description of the 323 cities included in the analysis.

	Arid climate zone (N = 79)	Non-arid climate zone (N = 244)
	Median [Interquartile Range] or N (%)	
Heat excess death fraction^2^	0.68% [-0.06, 1.67]	0.56% [-0.06, 1.58]
Number (%) of cities with Heat excess death fractions < 0	26 (30%)	74 (30%)
Average daily mean temperature(Degrees Celsius)	18.67 [16.49, 22.03]	21.36 [18.65, 23.88]
Optimal mortality temperature (Degrees Celsius)^1^	22.38 [20.74, 25.14]	23.89 [22.33, 25.16]
Annual Average PM_2.5_ concentration (µg/m^3^)	12.55 [8.04, 15.14]	10.35 [7.60, 12.95]
Social environment (unitless)^3^	0.27 [-0.15, 0.60]	0.04 [-0.36, 0.44]
Population weighted greenness (NDVI, unitless)^4^	0.31 [0.23, 0.38]	0.49 [0.46, 0.53]
Green space clustering^5^	0.80 [0.78, 0.84]	0.84 [0.80, 0.86]
Pearson correlation coefficient representing relationship between NDVI and clustering	−0.09 (p = 0.4)	−0.01 (p = 0.9)

Abbreviations: NDVI, normalized difference vegetation index; PM_2.5_, Particulate matter < 2.5 ug/m^3^.

1 The city-specific optimal mortality temperature was derived from distributed lag non-linear models of city-specific associations between daily ambient temperature and all-cause mortality counts. The optimal temperature was identified as the temperature (Degrees Celsius) at which heat-mortality risk was lowest.

2 Defined as the total deaths attributable to temperatures higher than optimal; expressed as a percentage.

3 Lower values of the index represent greater levels of social deprivation.

4 Higher values represent higher levels of greenness.

5 Higher values represent higher clustering and lower values represent lower clustering.

We stratified the meta-regression analyses by arid climate zone (arid vs. non-arid). We decided to stratify the analyses by climate zone for several reasons. First, the distribution of greenness varies substantially in the different regions, which creates obstacles, from a statistical perspective, when running analyses for all cities combined. For example, the median (interquartile range [IQR]) of NDVI in the arid climate zone cities was 31 (IQR: 0.23, 0.38) and 49 (IQR: 0.46, 0.53) in non-arid cities (Table 1). Second, the height and other features of vegetation vary according to climate zone, with dry climate zones having shorter vegetation with less expansive canopies (e.g., cacti and succulents) compared to more humid areas. This has implications for the cooling mechanisms of the different green spaces and for urban–rural temperature differentials (i.e., urban heat island effect). (Manickathan et al., 2018). Third, prior evidence shows different impacts of green space on thermal comfort in arid vs. non-arid climate zones (Jendritzky et al., 2012). We combined temperate and tropical zone cities for the following reasons: (1) to improve statistical power, (2) because in our data, the distribution of greenness was similar in both climate zones (Appendix 6), and (3) because in preliminary analyses, estimates of association of heat EDFs with greenness were similar in temperate and tropical climate zones.

#### 2.6.1 Interaction

To estimate effect modification of associations between greenness and green space clustering, we included an interaction term between the three-level categorical greenness variable and the dichotomous clustering term. We assessed statistical evidence of effect modification on the multiplicative scale by conducting likelihood ratio tests (LRT, two degrees of freedom).

#### 2.6.2 Additional analyses

As a complementary strategy to estimating associations with heat EDFs and to explore how greenness is associated with the relative risk in mortality associated with higher temperatures, we repeated all the analyses, replacing heat EDF with heat RR as the dependent variables. In addition, because population density may confound estimates of the association between green space and the heat-EDFs, as a sensitivity analysis, we adjusted the main analysis models for population density (entered into models as a continuous term). Finally, as an additional sensitivity analysis, we repeated the main analyses after excluding cities with negative heat EDFs.

All analyses were performed in R (version 3.6.0). (R Core Team, 2021) Modeling was performed using the mvmeta and dlmn packages (Gasparrini, 2011, Gasparrini et al., 2012).

## 3 Results

Table 1 presents descriptive statistics on the cities included in the analysis, stratified by climate zone. The city-specific heat-mortality curves, EDFs and RRs have been published previously (Kephart et al., 2022) and are viewable through a web-based application (https://drexel-uhc.shinyapps.io/MS85). Heat EDFs were higher in arid climate zones (Median: 0.68% in arid cities vs. 0.56% in non-arid cities). Approximately 30% of cities in both arid and non-arid climate zones had EDFs < 0. Cities in arid and non-arid climate zones were similar with respect to average daily mean temperature, “optimal” temperature, and greenspace clustering levels. Median PM_2.5_ concentrations were modestly higher in arid than non-arid climate zone cities (Median: 12.55 µg/m^3^ in arid cities and 10.35 µg/m^3^ in non-arid cities). Population weighted greenness was higher in temperate and tropical cities than arid ones (Median: 0.49 in non-arid cities and 0.31 in arid cities, map shown in Appendix 8). The social environment index variable was smaller in non-arid cities, suggesting greater social deprivation as compared to arid cities (Median: 0.04 in non-arid cities, and 0.27 in arid cities). Green space clustering was not correlated with overall greenness, based on relatively small correlation coefficients in non-arid and arid climate zone cities (Pearson correlation coefficient: −0.09 [p = 0.4] and −0.01 [p = 0.9], respectively).

### 3.1 Meta regression estimates of association between greenness and EDFs from heat

In cities located in arid climate zones, heat EDFs were smaller in association with incrementally moderate and high greenness levels, although the confidence intervals associated with the meta-estimates were wide and crossed the null, and we did not observe a dose–response relationship (Table 2). For example, compared to cities with the lowest levels of greenness, cities with moderate and high greenness levels had 0.41 (Beta: −0.41, 95% CI: −1.06, 0.25) and 0.23 (Beta: −0.23, 95% CI: −0.95, 0.49) percentage point lower heat EDFs in association with non-optimally high ambient temperatures, respectively. In non-arid climate zone cities, associations between greenness and the heat-EDFs were approximately null. We observed evidence of heterogeneity in the estimates of association across the cities, in both non arid and arid climate zones (p value associated with the Cochran’s Q test statistic < 0.01).

**Table 2:** Estimates of association between greenness, measured using the normalized difference vegetation index (NDVI), and heat excess death fractions, stratified by climate zone.

	**Arid climate zone cities** **(N = 79)**				**Temperate and tropical climate zone cities** **(N = 244)**			
**Greenness (NDVI)**	**Beta**	**95% CI**	**AIC**	**Cochran’s Q (p - value)**	**Beta**	**95% CI**	**AIC**	**Cochran’s Q** **(p -value)**
**Low**	**REF**				**REF**			
Moderate	−0.41	−1.06, 0.25	269.8	165.4 (<0.01)	−0.04	−0.25, 0.17	769.3	565.1 (<0.01)
High	−0.23	−0.95, 0.49			0.02	−0.22, 0.27		

Abbreviations: AIC, Akaike Information criterion; CI, confidence interval; EDF, excess death fractions; NDVI, normalized difference vegetation index.

1The reference category is cities in the lowest tertile of the climate-zone specific distribution of greenness, as measured by NDVI. Moderate and high levels of greenness refer to the second and third tertiles of the climate zone specific distribution of greenness, measured by the NDVI. The results were derived from random effects *meta*-regression models that were adjusted for particulate matter < 2.5 µg/m^3^, social environment index, country group, and a green space clustering metric. Models were run separately for arid and non-arid climate zone cities.

When we repeated analyses using the heat-RR as the dependent variable, all estimates were approximately null (Appendix 9). Additional adjustment for population density did not markedly change the estimates of association (Appendix 10). Also, rerunning analyses after excluding cities with EDFs < 0 did not markedly change the results (Appendix 11).

### 3.2 Interaction with clustering

We did not observe strong evidence of effect modification by clustering in arid or non-arid climate zones (p-values associated with the LRT > 0.10). However, based on the magnitude of the stratified estimates of association, in arid climate zones, the modest protective associations between higher greenness and the heat EDFs were restricted, primarily, to cities where green space clustering was low. For example, among arid cities with lower levels of green space clustering, those with the highest greenness levels had 0.67 percentage point lower heat-EDFs compared to cities with the lowest greenness levels, although the associated CI was wide and crossed the null (Beta: −0.67, 95% CI: −1.66, 0.33, Table 3).

**Table 3:** Estimates of association between greenness, measured using the normalized difference vegetation index (NDVI), and heat excess death fractions, stratified by climate zone and level of green space clustering.^1^

	**Low Green Space Clustering**			**High Green Space Clustering**					
**Arid climate zone cities (N = 79)**									

**Greenness**	**Beta**	**95% CI**		**Beta**	**95% CI**		**AIC**	**p for LRT**^2^	**Cochran’s Q**

Moderate	−0.40	−1.53	0.73	−0.33	−1.11	0.46	269.8	0.13	161.7
High	−0.67	−1.66	0.33	0.27	−0.74	1.27			

**Temperate and tropical climate zone cities (N = 244)**									
**Greenness**	**Beta**	**95% CI**		**Beta**	**95% CI**		**AIC**	**p for LRT**^2^	**Cochran’s Q**
Moderate	−0.12	−0.46	0.23	0.00	−0.27	0.27	775.5	0.34	550.1 (<0.01)
High	0.02	−0.37	0.40	0.02	−0.28	0.33			

Abbreviations: AIC, Akaike Information criterion; CI; confidence interval’; NDVI, normalized difference vegetation index.

1 The reference category is cities in the lowest tertile of the climate-zone specific distribution of greenness, as measured by NDVI. Moderate and high levels of greenness refer to the second and third tertiles of the climate zone specific distribution of greenness, measured by the NDVI. The results were derived from random effects *meta*-regression models that were adjusted for particulate matter < 2.5 µg/m^3^, social environment index, country group, and a measure of green space clustering. Models were run separately for arid and non-arid climate zones. Effect modification by green space clustering was assessed by including an interaction term between the three-level categorical greenness variable (NDVI) and a term representing clustering, dichotomized at the median of the climate-zone specific distribution. Clustering was measured using the FRAGSTATS 4.2 program, (McGarigal and Marks, 1995) and computed based on the spatial adjacency matrix of green patches. The clustering metric ranges between −1 and 1, where −1 indicates maximum disaggregation, 0 indicates random distribution, and 1 indicates maximum clustering.

2 The p-value for a likelihood ratio test (LRT) derives from a two-degree of freedom chi-square distributed test of improvement in model fit following inclusion of an interaction term between the three-level NDVI term and the two-level clustering term.

By contrast, among arid cities with higher levels of green space clustering, the association between high greenness and heat-EDFs and on the opposite side of the null (Beta: 0.27, 95% CI: −0.74, 1,27). In cities located in non-arid climate zones, associations between greenness and the heat EDFs were close to the null, regardless of the level of green space clustering.

## 4 Discussion

Cities across the world are investing in green infrastructure for heat mitigation and adaptation purposes. Little is known about whether higher greenness levels protect against heat-related death in Latin American cities, and whether associations vary according to climate zone or green space configuration. Results from this analysis of 323 Latin American cities suggest that higher city-level greenness may be associated with modest protection against heat-related deaths in arid climate zones, although the strength of evidence was weakened by the lack of a dose–response relationship and because estimates of association were imprecise and crossed the null.

Results from previous research on associations between greenness and heat-related mortality are inconsistent, though, overall, they suggest that higher amounts of green vegetation provides modest protection against heat-related mortality. (Schinasi et al., 2018); (Denpetkul and Phosri, 2021); (Son et al., 2016); (Sera et al., 2019) To date, the largest study of associations between green vegetation and heat-related mortality associations was an analysis of 452 locations (metropolitan areas, provinces, larger areas) in 24 countries including Europe, North America, Central America, and East Asia. (Sera et al., 2019) The analysis found that one interquartile range increase in NDVI was associated with 6.6% (95% CI: 0.1%, 12.6%) lower heat-mortality relative risk. (Choi et al., 2022) This evidence is somewhat consistent with our finding of a protective association with greenness in cities located in arid climate zones, although the protective estimate of associations that we observed was closer to the null and not statistically significant. We did not observe similar protective associations in cities located in tropical and temperate climate zones.

To our knowledge, ours is the first multi-city analysis of associations between greenness and heat-mortality outcomes to stratify by climate zone. Our results suggested that greenness could be associated with protection against excess deaths from heat in arid climate zones. This finding is consistent with evidence from exposure studies, which have suggested that vegetation has greater cooling capacity in hot and dry climates as compared with humid climate zones. (Zhou et al., 2017) This may be explained by negative correlations between relative humidity and evapotranspiration (i.e., transfer of water from land surfaces to the atmosphere) (Evapotranspiration, 2011, Farhat, 2018, Water Science School. Evapotranspiration and the water cycle, 2018), and thus, urban green vegetation cooling capacity. However, the estimates of association in our analysis were fairly modest and we did not find greater protective associations in association with higher greenness levels. Furthermore, the associated CIs crossed the null. As a result, results from our study should be interpreted with caution.

In addition to stratifying by arid climate zone, we explored modification of associations between greenness and heat EDFs by green space clustering. We did not observe statistical evidence of effect modification by clustering, although the magnitude of the estimates suggested that greenness may have stronger protective associations with heat related death in arid cities where green spaces are less clustered. Consistent with this finding, some (but not all) exposure studies have found that higher clustering of vegetation is associated with hotter land surface temperatures (Zhou et al., 2011, Kim et al., 2016). For example, in Phoenix, Arizona, more dispersed (and less clustered) green patches had greater regional cooling effects, while more clustered green patches were associated with enhanced cooling at a highly localized level; this suggests that the implications of configuration for cooling may vary according to spatial scale (Zhang et al., 2017). Results from other exposure studies do not support our findings, because they found that higher levels of clustering were associated with cooler land surface temperatures (Masoudi and Tan, 2019, Kim et al., 2016, Zhibin et al., 2015). In Singapore, larger green patches that were more connected and less fragmented were associated with lower land surface temperatures (Masoudi and Tan, 2019). In Changchun, China, greater aggregation and cohesion of green patches was associated with lower land surface temperatures (Zhibin et al., 2015). The associations observed in our analysis may be explained not just by greater overall cooling, but also by human interaction with green spaces. For examples, lower levels of green space clustering may correspond to more evenly distributed vegetation throughout a city, and thus to more even distribution of the associated cooling effects (Zhou et al., 2011). More uniform distribution of green spaces within a city may be important within a human context, because this may enhance opportunities for human interaction with green spaces (Zhou et al., 2011).

Our analysis has several strengths, including the use of a state-of-the-art green space configuration metric, application of robust statistical methods, inclusion of a large sample of cities from an understudied region, and adjustment for a variety of city-level covariates that might confound associations. Despite these strengths, we acknowledge several limitations. We lacked detailed information on green space type (e.g., trees vs. shrubs). Although we used population-weighted greenness measures, we lacked data on proximity of green space measures to peoples’ homes, which may influence the extent to which residents benefit from the cooling effects of green spaces. We used satellite imagery data from year 2017 to identify green patches and quantify green space spatial configuration. We used satellite images from this year because they allowed identification of high spatial resolution green patches. Nevertheless, there is a temporal mismatch between the mortality and green space configuration data, which could have resulted in misclassification of the clustering variable. In addition, because there is substantial variation across cities in terms of the timing and length of the “hot” season, we used the maximum NDVI value (within each pixel) to ascertain the greenest values. However, the greenest pixel level values may not always correspond to the hottest months. In addition, it is possible that our temperature data may already be capturing the cooling effect of green spaces, even at the 9 km horizontal resolution. This concern may be most pronounced for very large green spaces, which are more likely to alter measured temperature at such a coarse resolution (Aram et al., 2019). Further, despite adjusting for a number of city-level characteristics that might confound the meta-regression estimates, we lacked sufficient data to account for adaptive behaviors, resources, and/or biologic variables which may impact heat vulnerability, such as air conditioning access or use, housing conditions (e.g., building height, position, insulation, and construction materials); and underlying chronic conditions within the population (Samuelson et al., 2020, Reid et al., 2009). Also, in this city-level analysis, we did not account for intra-urban differences in temperatures and micro-urban heat islands, which are known to exist (Harlan et al., 2006). We also lacked data on wind speed and humidity levels, which may contribute to human thermal comfort (Yin et al., 2012). We were therefore unable to adjust for these factors at either the first- or second- stages of the analysis. Another limitation is that our results may not generalize to some other cities in Latin America, which may differ from the ones included in this study in terms of level of greenness or other urban characteristics that may impact heat vulnerability.

Despite these limitations, our work represents a critical contribution to the knowledge base about green space as an urban heat mitigation and adaptation strategy. There is a need for further research on the impact of land cover configuration on heat vulnerability in different study settings, especially in LMIC, and to explore these impacts and relationships at the micro-urban neighborhood level. There is also a critical need for further research on the types of green space that may be most important for reducing heat vulnerability.

## 5 Conclusions

Within the context of the earth’s rapidly and dangerously warming climate, urban greening is a promising and attractive intervention strategy for heat mitigation and adaptation. Results from our work provide preliminary evidence to suggest that green interventions may be useful as a heat adaptation strategy, particularly in arid climate zone Latin American cities. Our results also suggest that the configuration of green spaces within cities may be important. However, given that any estimates of association observed in this study were modest in magnitude and had wide CIs, this work should be replicated in other settings. Further research is also needed to explore the extent to which relationships vary according to different types of green space cover, green space configuration, and/or across climate zones.

## CRediT authorship contribution statement

**Leah H Schinasi:** Conceptualization, Formal analysis, Methodology, Writing – original draft. **Maryia Bakhtsiyarava:** Formal analysis, Writing – review & editing, Methodology, Visualization. **Brisa N. Sanchez:** Methodology, Software, Writing – review & editing. **Josiah L. Kephart:** Writing – review & editing. **Yang Ju:** Data curation, Writing – review & editing. **Sarav Arunachalam:** Data curation, Writing – review & editing. **Nelson Gouveia:** Writing – review & editing. **Waleska Teixeira Caiaffa:** Writing – review & editing. **Marie S. O'Neill:** Writing – review & editing. **Iryna Dronova:** Data curation, Writing – review & editing, Conceptualization. **Ana V. Diez Roux:** Funding acquisition, Conceptualization, Writing – review & editing. **Daniel A. Rodriguez:** Funding acquisition, Conceptualization, Writing – review & editing.

## Declaration of Competing Interest

The authors declare that they have no known competing financial interests or personal relationships that could have appeared to influence the work reported in this paper.

## Data Availability

Data will be made available on request.
