# Supplementary material for: Greenness and excess deaths from heat in 323 Latin American cities: Do associations vary according to climate zone or green space configuration?
Source: Environ Int. 2023 Oct;180:108230. doi: 10.1016/j.envint.2023.108230 (PMC10594062; doi:10.1016/j.envint.2023.108230)
Supplement: Supplementary data 1 [file mmc1.docx]

| **Appendix 1. Description of the green space configuration metrics.** | |
| --- | --- |
| **Green space measure** | **Description** |
| Population weighted mean of annual maximum NDVI values | Density of vegetation, or overall greenness. This unitless index ranges in value from -1 to 1. Negative values represent cloud and water, values close to 0 indicate barren rock and soil, and values close to 1 represent the highest densities of vegetation. |
| Patch density (# patches/hectare) | Number of green patches divided by total unit area. Higher patch density indicates more patches. When we hold % greenspace constant, a higher value suggests greater fragmentation of patches. |
| Clumpiness of green space patches (unitless) | Describes the extent to which greenspace is clustered (or clumped) given its total area within the city. Clumpiness is computed based on the spatial adjacency matrix of green patches, which shows the frequency with which different pairs of patches appear side-by-side in space. This variable ranges between -1 and 1, where -1 indicates maximum disaggregation, 0 indicates random distribution, and 1 indicates maximum aggregation. |
| Mean nearest neighbor distance of green space patches (meters) | The mean of Euclidean nearest neighbor distance (ENN) across all green patches. Describes how far, on average, green patches are located from one another. A higher value represents greater isolation of green space patches from one another. |

**Appendix 2. Pearson correlation coefficients representing relationships between greenness (NDVI), the green space patch metrics, % built up and population density, stratified by climate zone; ***p< 0.001, **p < 0.01, *<0.05.**

**
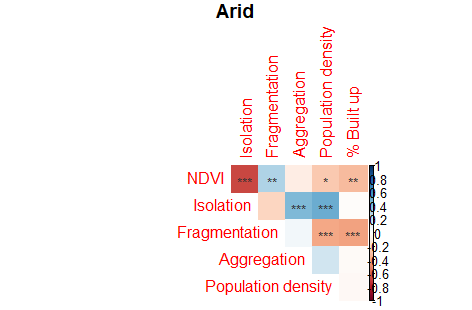
**

**
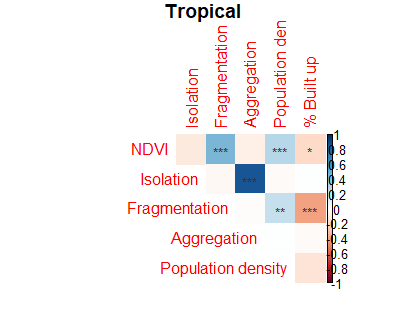
**


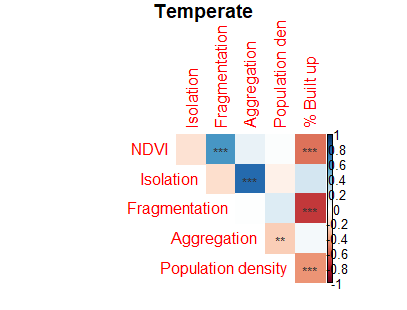


**Appendix 3. Maps illustrating green space clustering in two cities with high and low clumpiness indices.***


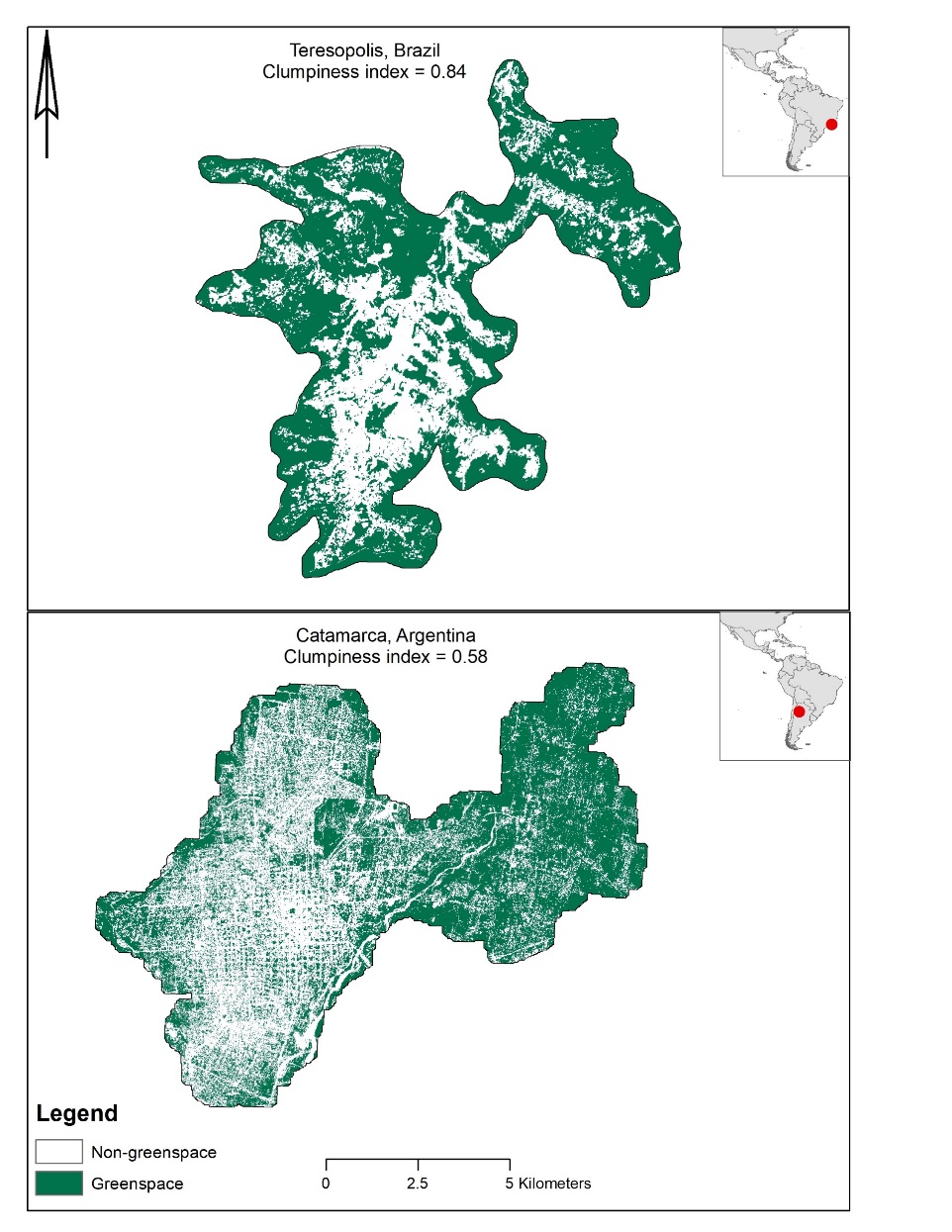


*Each green pixel represents a 30x30 meter patch of greenspace derived from the land cover green space map described in the main text. The two cities have comparable levels of green space as measured by % green (green space occupies 55% of Catamarca and 65% of Teresopolis city area) but vary in the clustering patterns of green space: Catamarca has the lowest levels of green space clustering among the cities, whereas Teresopolis has one of the highest.

**Appendix 4. Distributed lag (0-21 days) nonlinear conditional Poisson models to estimate associations between mean daily temperature and all-cause mortality for each city**

Y_tc_ ~ Poisson(μ_tc_) Log (μ_tc_) = α + s(T_t,c_; θc ) +  λStrata_t_

where t is the day of the observation in city *c*; Y_tc_ is the observed daily number of deaths on day t in city c; α is the intercept; T_tc_ is average daily temperature measured on day t in city c and all preceding days up to lag 21, modeled as a distributed lag nonlinear function and natural cubic spline with knots placed at the minimum, maximum, and 10^th^, 75^th^, and 90^th^ percentiles of the city-specific distribution of average daily temperatures and the lag function was modeled as natural cubic B spline with an intercept and three internal knots place at intervals spaced at equivalent amounts from one another on the log scale; and Strata_t_ is a categorical variable for the day of the week, month, and year of the study (used to control for seasonality and long-term temporal trends).

**Appendix 5. Latin American cities included in the analysis.**

| City | Country | Climate zone | Average daily temperature (Degrees Celsius) | Minimum mortality temperature (Degrees Celsius) |
| --- | --- | --- | --- | --- |
| San Fernando del Valle de Catamarca | Argentina | Arid | 18.59 | 24.29 |
| Comodoro Rivadavia | Argentina | Arid | 11.84 | 17.97 |
| La Rioja | Argentina | Arid | 18.4 | 24.6 |
| San Rafael | Argentina | Arid | 15.85 | 23.17 |
| Neuquén-Plottier-Cipolletti | Argentina | Arid | 15.45 | 23.15 |
| Santiago del Estero- La Banda | Argentina | Arid | 21.76 | 27.33 |
| San Juan | Argentina | Arid | 17.12 | 22.62 |
| Rawson - Trelew | Argentina | Arid | 14.23 | 20.97 |
| Río Gallegos | Argentina | Arid | 6.79 | 16.73 |
| Petrolina | Brazil | Arid | 26.39 | 27.19 |
| Arica | Chile | Arid | 17.38 | 21.42 |
| Iquique | Chile | Arid | 17.21 | 21.90 |
| Antofagasta | Chile | Arid | 13.51 | 17.86 |
| Calama | Chile | Arid | 12.94 | 17.61 |
| Copiapó | Chile | Arid | 17.00 | 18.77 |
| La Serena-Coquimbo | Chile | Arid | 16.38 | 21.16 |
| Chimbote | Peru | Arid | 19.54 | 22.51 |
| Arequipa | Peru | Arid | 13.55 | 17.19 |
| Ayacucho | Peru | Arid | 11.54 | 14.00 |
| Chincha Alta | Peru | Arid | 19.86 | 21.34 |
| Ica | Peru | Arid | 21.29 | 22.61 |
| Pisco (incl. San Clemente) | Peru | Arid | 19.89 | 22.38 |
| Chiclayo | Peru | Arid | 21.34 | 23.78 |
| Lima | Peru | Arid | 18.86 | 21.07 |
| Trujillo | Peru | Arid | 19.53 | 20.80 |
| Piura | Peru | Arid | 23.81 | 25.67 |
| Sullana | Peru | Arid | 23.86 | 25.53 |
| Tacna | Peru | Arid | 17.37 | 19.27 |
| Tumbes | Peru | Arid | 24.50 | 25.32 |
| Aguascalientes (ZM de Aguascalientes) | Mexico | Arid | 18.02 | 20.40 |
| Ensenada | Mexico | Arid | 17.01 | 22.36 |
| Mexicali (ZM de Mexicali) | Mexico | Arid | 24.49 | 33.47 |
| La Paz | Mexico | Arid | 23.69 | 26.52 |
| Chihuahua (ZM de Chihuahua) | Mexico | Arid | 18.54 | 22.90 |
| Ciudad Juárez (Juárez) (ZM de Juárez) | Mexico | Arid | 18.37 | 24.96 |
| Cuauhtémoc | Mexico | Arid | 14.30 | 18.8 |
| Delicias | Mexico | Arid | 20.86 | 24.88 |
| Hidalgo del Parral | Mexico | Arid | 17.49 | 21.45 |
| Ciudad Acuña | Mexico | Arid | 21.70 | 26.19 |
| Monclova (ZM Monclova-Frontera) | Mexico | Arid | 21.31 | 24.85 |
| Piedras Negras (ZM Piedras Negras) | Mexico | Arid | 22.6 | 26.87 |
| Saltillo (ZM de Saltillo) | Mexico | Arid | 16.68 | 19.54 |
| Torreón (ZM de la Laguna) | Mexico | Arid | 22.31 | 24.97 |
| Colima (ZM Colima-Villa de Álvarez) | Mexico | Arid | 23.13 | 23.90 |
| Manzanillo | Mexico | Arid | 24.99 | 25.48 |
| Tecomán (ZM Tecomán) | Mexico | Arid | 25.54 | 25.82 |
| Celaya (ZM de Celaya) | Mexico | Arid | 18.39 | 20.63 |
| Irapuato | Mexico | Arid | 19.09 | 21.36 |
| León (León de los Aldama) (ZM de León) | Mexico | Arid | 18.42 | 20.92 |
| Salamanca | Mexico | Arid | 19.40 | 21.50 |
| San Francisco del Rincón (ZM de San Francisco del Rincón) | Mexico | Arid | 18.67 | 20.98 |
| Pachuca (Pachuca de Soto) (ZM de Pachuca) | Mexico | Arid | 13.22 | 18.37 |
| Tula de Allende (ZM de Tula) | Mexico | Arid | 15.33 | 21.20 |
| La Piedad (La Piedad de Cabadas) (ZM de La Piedad-Pénjamo) | Mexico | Arid | 18.87 | 21.19 |
| Zamora de Hidalgo (ZM de Zamora-Jacona) | Mexico | Arid | 18.98 | 20.90 |
| Cuautla (ZM de Cuautla) | Mexico | Arid | 20.92 | 22.64 |
| Cuernavaca (ZM de Cuernavaca) | Mexico | Arid | 19.65 | 21.06 |
| Monterrey (ZM de Monterrey) | Mexico | Arid | 20.95 | 23.70 |
| Querétaro (Santiago de Querétaro) (ZM de Querétaro) | Mexico | Arid | 17.51 | 19.71 |
| San Juan del Río | Mexico | Arid | 16.51 | 18.49 |
| Culiacán (Culiacán Rosales) | Mexico | Arid | 24.53 | 25.62 |
| Los Mochis (Ahome) | Mexico | Arid | 24.78 | 27.73 |
| Rioverde (Río Verde) (ZM de Río Verde-Ciudad Fernández) | Mexico | Arid | 19.64 | 21.72 |
| San Luis Potosí (ZM de San Luis Potosí-Soledad de Graciano Sánchez) | Mexico | Arid | 16.46 | 18.76 |
| Ciudad Obregón (Cajeme) | Mexico | Arid | 24.78 | 28.35 |
| Guaymas (Heroica Guaymas) (ZM de Guaymas) | Mexico | Arid | 24.37 | 29.71 |
| Hermosillo | Mexico | Arid | 24.20 | 30.92 |
| Navojoa | Mexico | Arid | 24.87 | 27.83 |
| Nogales (Heroica Nogales) | Mexico | Arid | 17.38 | 23.11 |
| San Luis Río Colorado | Mexico | Arid | 24.08 | 32.32 |
| Ciudad Victoria | Mexico | Arid | 21.57 | 23.93 |
| Nuevo Laredo (ZM de Nuevo Laredo) | Mexico | Arid | 23.76 | 26.60 |
| Reynosa (ZM de Reynosa-Río Bravo) | Mexico | Arid | 23.90 | 25.93 |
| Fresnillo | Mexico | Arid | 16.14 | 18.91 |
| Zacatecas (ZM de Zacatecas-Guadalupe) | Mexico | Arid | 15.53 | 18.25 |
| Córdoba | Argentina | Temperate | 16.91 | 22.24 |
| Río Cuarto | Argentina | Temperate | 16.92 | 22.91 |
| Resistencia | Argentina | Temperate | 21.43 | 26.62 |
| Corrientes | Argentina | Temperate | 21.39 | 26.17 |
| Buenos Aires | Argentina | Temperate | 17.29 | 23.49 |
| Concordia | Argentina | Temperate | 18.95 | 24.83 |
| Paraná | Argentina | Temperate | 18.69 | 24.66 |
| Formosa | Argentina | Temperate | 22.47 | 26.69 |
| San Salvador de Jujuy | Argentina | Temperate | 14.90 | 17.74 |
| Santa Rosa-Toay | Argentina | Temperate | 16.24 | 23.81 |
| Posadas | Argentina | Temperate | 21.34 | 25.36 |
| San Carlos de Bariloche | Argentina | Temperate | 6.22 | 18.84 |
| Salta | Argentina | Temperate | 15.73 | 19.02 |
| Rosario | Argentina | Temperate | 18.08 | 24.5 |
| Santa Fe | Argentina | Temperate | 19.08 | 25.07 |
| San Luis | Argentina | Temperate | 17.22 | 24.44 |
| Villa Mercedes | Argentina | Temperate | 16.82 | 23.93 |
| San Miguel de Tucumán-Tafí Viejo | Argentina | Temperate | 18.30 | 23.77 |
| Barbacena | Brazil | Temperate | 18.31 | 20.88 |
| Conselheiro Lafaiete | Brazil | Temperate | 19.04 | 21.54 |
| Juiz de Fora | Brazil | Temperate | 19.98 | 22.93 |
| Poços de Caldas | Brazil | Temperate | 18.11 | 20.56 |
| Pouso Alegre | Brazil | Temperate | 19.52 | 22.36 |
| Varginha | Brazil | Temperate | 19.91 | 22.31 |
| Apucarana | Brazil | Temperate | 20.31 | 22.53 |
| Arapongas | Brazil | Temperate | 20.47 | 23.12 |
| Cascavel | Brazil | Temperate | 19.91 | 22.86 |
| Curitiba | Brazil | Temperate | 17.20 | 19.92 |
| Foz do Iguaçu | Brazil | Temperate | 22.21 | 25.48 |
| Guarapuava | Brazil | Temperate | 17.17 | 19.70 |
| Londrina | Brazil | Temperate | 20.98 | 23.01 |
| Maringá | Brazil | Temperate | 21.79 | 23.86 |
| Paranaguá | Brazil | Temperate | 21.09 | 24.29 |
| Ponta Grossa | Brazil | Temperate | 18.07 | 21.48 |
| Toledo | Brazil | Temperate | 21.00 | 23.05 |
| Angra dos Reis | Brazil | Temperate | 21.49 | 24.55 |
| Nova Friburgo | Brazil | Temperate | 18.33 | 21.58 |
| Petrópolis | Brazil | Temperate | 19.45 | 22.54 |
| Teresópolis | Brazil | Temperate | 18.67 | 22.14 |
| Caxias do Sul | Brazil | Temperate | 16.68 | 19.74 |
| Passo Fundo | Brazil | Temperate | 17.83 | 20.70 |
| Pelotas | Brazil | Temperate | 18.33 | 23.23 |
| Porto Alegre | Brazil | Temperate | 19.28 | 23.75 |
| Rio Grande | Brazil | Temperate | 18.45 | 23.07 |
| Santa Cruz do Sul | Brazil | Temperate | 18.78 | 23.27 |
| Santa Maria | Brazil | Temperate | 18.89 | 23.71 |
| Uruguaiana | Brazil | Temperate | 19.92 | 25.40 |
| Balneário Camboriú | Brazil | Temperate | 20.39 | 23.92 |
| Blumenau | Brazil | Temperate | 19.44 | 23.09 |
| Brusque | Brazil | Temperate | 19.41 | 23.31 |
| Chapecó | Brazil | Temperate | 18.90 | 22.16 |
| Criciúma | Brazil | Temperate | 19.52 | 23.72 |
| Florianópolis | Brazil | Temperate | 20.25 | 23.78 |
| Itajaí | Brazil | Temperate | 20.70 | 24.25 |
| Jaraguá do Sul | Brazil | Temperate | 19.84 | 23.65 |
| Joinville | Brazil | Temperate | 20.56 | 24.18 |
| Lages (Lajes) | Brazil | Temperate | 15.90 | 19.12 |
| Atibaia | Brazil | Temperate | 19.25 | 21.91 |
| Botucatu | Brazil | Temperate | 20.19 | 23.29 |
| Bragança Paulista | Brazil | Temperate | 19.42 | 22.33 |
| Guaratinguetá | Brazil | Temperate | 20.43 | 23.24 |
| Itapetininga | Brazil | Temperate | 19.69 | 22.7 |
| Jundiaí | Brazil | Temperate | 19.45 | 21.86 |
| São José dos Campos | Brazil | Temperate | 20.05 | 22.65 |
| São Paulo | Brazil | Temperate | 19.29 | 22.39 |
| Sorocaba | Brazil | Temperate | 20.26 | 23.78 |
| Tatuí | Brazil | Temperate | 20.50 | 23.19 |
| Taubaté | Brazil | Temperate | 20.36 | 23.14 |
| Tubarao | Brazil | Temperate | 20.00 | 24.30 |
| Bento Goncalves | Brazil | Temperate | 17.15 | 21.29 |
| Caraguatatuba | Brazil | Temperate | 21.38 | 24.12 |
| Parobe | Brazil | Temperate | 18.44 | 22.70 |
| Valparaíso-Viña del Mar | Chile | Temperate | 14.13 | 19.56 |
| Quillota | Chile | Temperate | 15.04 | 18.42 |
| San Antonio | Chile | Temperate | 13.80 | 19.56 |
| Santiago de Chile | Chile | Temperate | 14.44 | 23.52 |
| Rancagua | Chile | Temperate | 12.35 | 18.91 |
| Talca | Chile | Temperate | 14.67 | 20.87 |
| Curicó | Chile | Temperate | 14.14 | 23.39 |
| Concepción | Chile | Temperate | 13.14 | 21.06 |
| Chillán | Chile | Temperate | 13.70 | 19.69 |
| Los Ángeles | Chile | Temperate | 13.16 | 19.46 |
| Temuco | Chile | Temperate | 11.51 | 17.31 |
| Valdivia | Chile | Temperate | 11.09 | 20.17 |
| Osorno | Chile | Temperate | 10.76 | 20.08 |
| Puerto Montt | Chile | Temperate | 10.28 | 18.06 |
| Cajamarca | Peru | Temperate | 11.59 | 13.58 |
| Cusco (Cuzco) | Peru | Temperate | 7.93 | 10.55 |
| Huánuco | Peru | Temperate | 13.98 | 16.00 |
| Huancayo | Peru | Temperate | 8.59 | 10.83 |
| Juliaca | Peru | Temperate | 8.22 | 12.16 |
| Quetzaltenango | CenAm | Temperate | 14.28 | 16.48 |
| Tijuana (ZM e Tijuana) | Mexico | Temperate | 16.31 | 21.88 |
| San Cristóbal de las Casas | Mexico | Temperate | 15.53 | 18.79 |
| Durango (Victoria de Durango) | Mexico | Temperate | 16.54 | 20.68 |
| Guanajuato | Mexico | Temperate | 17.25 | 19.97 |
| Uriangato (ZM de Moroleón-Uriangato) | Mexico | Temperate | 18.12 | 21.21 |
| Tulancingo (Tulancingo de Bravo) (ZM de Tulancingo) | Mexico | Temperate | 13.16 | 18.08 |
| Guadalajara (ZM de Guadalajara) | Mexico | Temperate | 20.07 | 22.77 |
| Ocotlán (ZM de Ocotlán) | Mexico | Temperate | 20.19 | 22.66 |
| Ciudad de México [Mexico City] (ZM del Valle de México) | Mexico | Temperate | 14.95 | 17.45 |
| Tianguistenco (ZM de Tianguistenco) | Mexico | Temperate | 12.21 | 16.46 |
| Toluca (Toluca de Lerdo) (ZM de Toluca) | Mexico | Temperate | 12.91 | 17.55 |
| Morelia (ZM de Morelia) | Mexico | Temperate | 16.52 | 18.39 |
| Uruapan (Uruapan del Progreso) | Mexico | Temperate | 17.73 | 19.15 |
| Oaxaca (Oaxaca de Juárez) (ZM de Oaxaca) | Mexico | Temperate | 17.71 | 19.74 |
| Puebla (Heróica Puebla de Zaragoza) (ZM Puebla) | Mexico | Temperate | 15.97 | 20.49 |
| Tehuacán (ZM de Tehuacán) | Mexico | Temperate | 17.23 | 19.42 |
| Teziutlán (ZM de Teziutlán) | Mexico | Temperate | 13.92 | 18.76 |
| Matamoros (Heroica Matamoros) (ZM de Matamoros) | Mexico | Temperate | 23.67 | 25.45 |
| Tlaxcala (Tlaxcala de Xicohténcatl) (ZM de Tlaxcala-Apizaco) | Mexico | Temperate | 14.11 | 18.98 |
| Orizaba (ZM de Orizaba) | Mexico | Temperate | 16.79 | 19.45 |
| Xalapa-Enríquez (Jalapa) (ZM de Xalapa) | Mexico | Temperate | 18.59 | 23.74 |
| Rio Branco | Brazil | Tropical | 25.45 | 25.78 |
| Arapiraca | Brazil | Tropical | 24.66 | 25.74 |
| Maceió | Brazil | Tropical | 25.20 | 25.73 |
| Macapá | Brazil | Tropical | 26.31 | 25.83 |
| Manaus | Brazil | Tropical | 26.29 | 25.36 |
| Alagoinhas | Brazil | Tropical | 24.51 | 25.20 |
| Barreiras | Brazil | Tropical | 25.00 | 24.23 |
| Feira de Santana | Brazil | Tropical | 24.42 | 25.31 |
| Ilhéus | Brazil | Tropical | 23.8 | 24.38 |
| Itabuna | Brazil | Tropical | 23.33 | 25.11 |
| Jequié | Brazil | Tropical | 22.69 | 23.55 |
| Porto Seguro | Brazil | Tropical | 24.11 | 24.69 |
| Salvador | Brazil | Tropical | 25.24 | 27.96 |
| Teixeira de Freitas | Brazil | Tropical | 23.88 | 24.83 |
| Vitória da Conquista | Brazil | Tropical | 20.95 | 22.61 |
| Fortaleza | Brazil | Tropical | 26.61 | 28.13 |
| Juazeiro do Norte | Brazil | Tropical | 25.91 | 26.76 |
| Sobral | Brazil | Tropical | 27.18 | 27.03 |
| Brasília | Brazil | Tropical | 21.74 | 23.37 |
| Cachoeiro de Itapemirim | Brazil | Tropical | 22.5 | 25.32 |
| Guarapari | Brazil | Tropical | 23.26 | 24.46 |
| Linhares | Brazil | Tropical | 23.83 | 25.05 |
| Vitória | Brazil | Tropical | 23.37 | 25.30 |
| Anápolis | Brazil | Tropical | 22.12 | 23.82 |
| Goiânia | Brazil | Tropical | 23.33 | 24.55 |
| Rio Verde | Brazil | Tropical | 23.20 | 24.97 |
| Caxias | Brazil | Tropical | 27.42 | 26.91 |
| Imperatriz | Brazil | Tropical | 26.89 | 27.42 |
| São Luís | Brazil | Tropical | 26.72 | 28.3 |
| Cuiabá | Brazil | Tropical | 26.25 | 26.42 |
| Rondonópolis | Brazil | Tropical | 25.88 | 26.61 |
| Campo Grande | Brazil | Tropical | 23.47 | 25.66 |
| Dourados | Brazil | Tropical | 23.31 | 24.76 |
| Araguari | Brazil | Tropical | 22.29 | 23.93 |
| Belo Horizonte | Brazil | Tropical | 20.52 | 22.00 |
| Divinópolis | Brazil | Tropical | 21.19 | 23.77 |
| Governador Valadares | Brazil | Tropical | 24.07 | 23.87 |
| Ipatinga | Brazil | Tropical | 22.66 | 24.42 |
| Itabira | Brazil | Tropical | 20.21 | 22.28 |
| Montes Claros | Brazil | Tropical | 23.00 | 25.15 |
| Passos | Brazil | Tropical | 21.24 | 22.90 |
| Patos de Minas | Brazil | Tropical | 21.62 | 23.21 |
| Sete Lagoas | Brazil | Tropical | 21.49 | 23.84 |
| Teófilo Otoni | Brazil | Tropical | 22.62 | 24.68 |
| Uberaba | Brazil | Tropical | 22.67 | 24.37 |
| Uberlândia | Brazil | Tropical | 22.18 | 24.14 |
| Belém | Brazil | Tropical | 26.51 | 28.71 |
| Castanhal | Brazil | Tropical | 26.23 | 25.80 |
| Marabá | Brazil | Tropical | 26.34 | 27.07 |
| Parauapebas | Brazil | Tropical | 25.75 | 25.71 |
| Santarém | Brazil | Tropical | 26.84 | 26.89 |
| Campina Grande | Brazil | Tropical | 23.49 | 25.90 |
| João Pessoa | Brazil | Tropical | 25.74 | 25.52 |
| Caruaru | Brazil | Tropical | 22.81 | 23.79 |
| Garanhuns | Brazil | Tropical | 21.75 | 23.03 |
| Recife | Brazil | Tropical | 25.55 | 25.98 |
| Vitória de Santo Antão | Brazil | Tropical | 24.18 | 24.47 |
| Parnaíba | Brazil | Tropical | 27.48 | 26.86 |
| Teresina | Brazil | Tropical | 27.82 | 27.52 |
| Araruama | Brazil | Tropical | 23.02 | 24.88 |
| Cabo Frio | Brazil | Tropical | 23.04 | 25.51 |
| Campos dos Goytacazes | Brazil | Tropical | 23.69 | 25.63 |
| Macaé | Brazil | Tropical | 22.96 | 24.09 |
| Resende | Brazil | Tropical | 20.57 | 23.34 |
| Rio de Janeiro | Brazil | Tropical | 23.05 | 23.33 |
| Volta Redonda | Brazil | Tropical | 21.00 | 23.31 |
| Mossoró | Brazil | Tropical | 27.75 | 27.25 |
| Natal | Brazil | Tropical | 25.90 | 25.04 |
| Ji-Paraná | Brazil | Tropical | 25.90 | 26.25 |
| Porto Velho | Brazil | Tropical | 26.01 | 24.80 |
| Boa Vista | Brazil | Tropical | 26.86 | 26.31 |
| Araçatuba | Brazil | Tropical | 23.87 | 25.01 |
| Araraquara | Brazil | Tropical | 22.01 | 23.78 |
| Araras | Brazil | Tropical | 21.24 | 23.68 |
| Santos | Brazil | Tropical | 21.66 | 23.57 |
| Barretos | Brazil | Tropical | 23.51 | 25.15 |
| Bauru | Brazil | Tropical | 22.08 | 23.96 |
| Birigui | Brazil | Tropical | 23.75 | 25.36 |
| Campinas | Brazil | Tropical | 20.94 | 24.03 |
| Catanduva | Brazil | Tropical | 23.07 | 24.49 |
| Franca | Brazil | Tropical | 21.24 | 23.47 |
| Jaú | Brazil | Tropical | 21.95 | 23.75 |
| Limeira | Brazil | Tropical | 21.44 | 24.41 |
| Marília | Brazil | Tropical | 22.40 | 24.24 |
| Mogi Guaçu (Moji Guaçu) | Brazil | Tropical | 21.32 | 23.61 |
| Ourinhos | Brazil | Tropical | 22.09 | 24.05 |
| Piracicaba | Brazil | Tropical | 21.50 | 23.96 |
| Presidente Prudente | Brazil | Tropical | 23.14 | 24.45 |
| Ribeirão Preto | Brazil | Tropical | 22.62 | 24.02 |
| Rio Claro | Brazil | Tropical | 21.33 | 24.35 |
| São Carlos | Brazil | Tropical | 20.88 | 23.43 |
| São José do Rio Preto | Brazil | Tropical | 23.26 | 24.70 |
| Sertãozinho | Brazil | Tropical | 23.04 | 25.01 |
| Aracaju | Brazil | Tropical | 25.35 | 25.91 |
| Araguaína | Brazil | Tropical | 26.38 | 25.83 |
| Palmas | Brazil | Tropical | 26.71 | 25.99 |
| Rio das Ostras | Brazil | Tropical | 22.95 | 25.32 |
| Tarapoto | Peru | Tropical | 24.12 | 24.34 |
| San José | CenAm | Tropical | 19.2 | 19.76 |
| San Miguel | CenAm | Tropical | 27.19 | 26.59 |
| San Salvador | CenAm | Tropical | 23.82 | 26.52 |
| Santa Ana | CenAm | Tropical | 23.21 | 23.81 |
| Escuintla | CenAm | Tropical | 23.57 | 24.25 |
| Ciudad de Guatemala | CenAm | Tropical | 18.57 | 20.15 |
| Campeche (San Francisco de Campeche) | Mexico | Tropical | 26.87 | 27.33 |
| Ciudad del Carmen | Mexico | Tropical | 26.44 | 25.9 |
| Tapachula (Tapachula de Córdova y Ordóñez) | Mexico | Tropical | 24.44 | 24.89 |
| Tuxtla Gutiérrez (ZM Tuxtla Gutiérrez) | Mexico | Tropical | 22.31 | 23.45 |
| Acapulco (Acapulco de Juárez) (ZM de Acapulco) | Mexico | Tropical | 25.80 | 25.07 |
| Chilpancingo (Chilpancingo de los Bravo) | Mexico | Tropical | 19.63 | 23.03 |
| Iguala (Iguala de la Independencia) | Mexico | Tropical | 25.78 | 24.21 |
| Puerto Vallarta (ZM de Puerto Vallarta) | Mexico | Tropical | 23.71 | 23.94 |
| Tepic (ZM de Tepic) | Mexico | Tropical | 20.42 | 21.55 |
| San Juan Bautista Tuxtepec | Mexico | Tropical | 25.06 | 26.34 |
| Santo Domingo Tehuantepec (ZM de Tehuantepec) | Mexico | Tropical | 26.29 | 25.76 |
| Cancún (ZM de Cancún) | Mexico | Tropical | 25.98 | 26.43 |
| Chetumal (Othón P. Blanco) | Mexico | Tropical | 25.80 | 25.61 |
| Playa del Carmen | Mexico | Tropical | 25.81 | 25.80 |
| Mazatlán | Mexico | Tropical | 24.30 | 25.26 |
| Ciudad Valles | Mexico | Tropical | 23.24 | 24.85 |
| Villahermosa (ZM de Villahermosa) | Mexico | Tropical | 26.18 | 25.05 |
| Tampico (ZM de Tampico) | Mexico | Tropical | 24.33 | 25.09 |
| Acayucan (ZM de Acayucan) | Mexico | Tropical | 24.84 | 25.46 |
| Coatzacoalcos (ZM de Coatzacoalcos) | Mexico | Tropical | 25.32 | 24.69 |
| Córdoba (ZM de Córdoba) | Mexico | Tropical | 19.02 | 20.45 |
| Minatitlán (ZM de Minatitlán) | Mexico | Tropical | 25.23 | 23.93 |
| Poza Rica de Hidalgo (ZM de Poza Rica) | Mexico | Tropical | 23.27 | 24.27 |
| Veracruz (ZM de Veracruz) | Mexico | Tropical | 25.10 | 25.13 |
| Mérida (ZM de Mérida) | Mexico | Tropical | 26.54 | 25.65 |
| Panama City | CenAm | Tropical | 25.57 | 25.41 |
| Colon | CenAm | Tropical | 25.54 | 27.31 |
| David | CenAm | Tropical | 24.84 | 25.49 |

| **Appendix 6. Tertiles of the distribution of population weighted normalized difference vegetation index (NDVI, unitless), stratified by climate zone.** | | | | |
| --- | --- | --- | --- | --- |
| Climate zone | Minimum | Tertile 1 | Tertile 2 | Tertile 3 |
| Arid | 0.11 | 0.26 | 0.35 | 0.54 |
| Temperate | 0.30 | 0.45 | 0.51 | 0.72 |
| Tropical | 0.36 | 0.48 | 0.51 | 0.70 |
| Non-arid (Temperate and tropical combined) | 0.30 | 0.47 | 0.51 | 0.72 |

**Appendix 7. Directed Acyclic Graph representing associations between greenness, heat-related mortality, and other city-level covariates.**


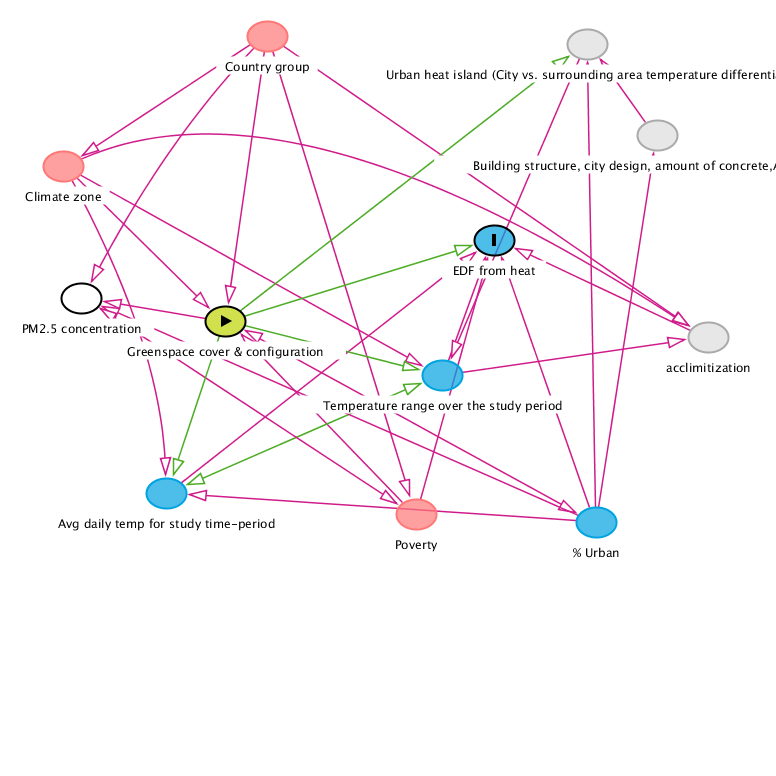


**Appendix 8. Spatial locations and overall greenness, measured as the average of the median of the maximum annual population weighted normalized difference vegetation index across the study years, in the 323 cities included in the analysis.**

**
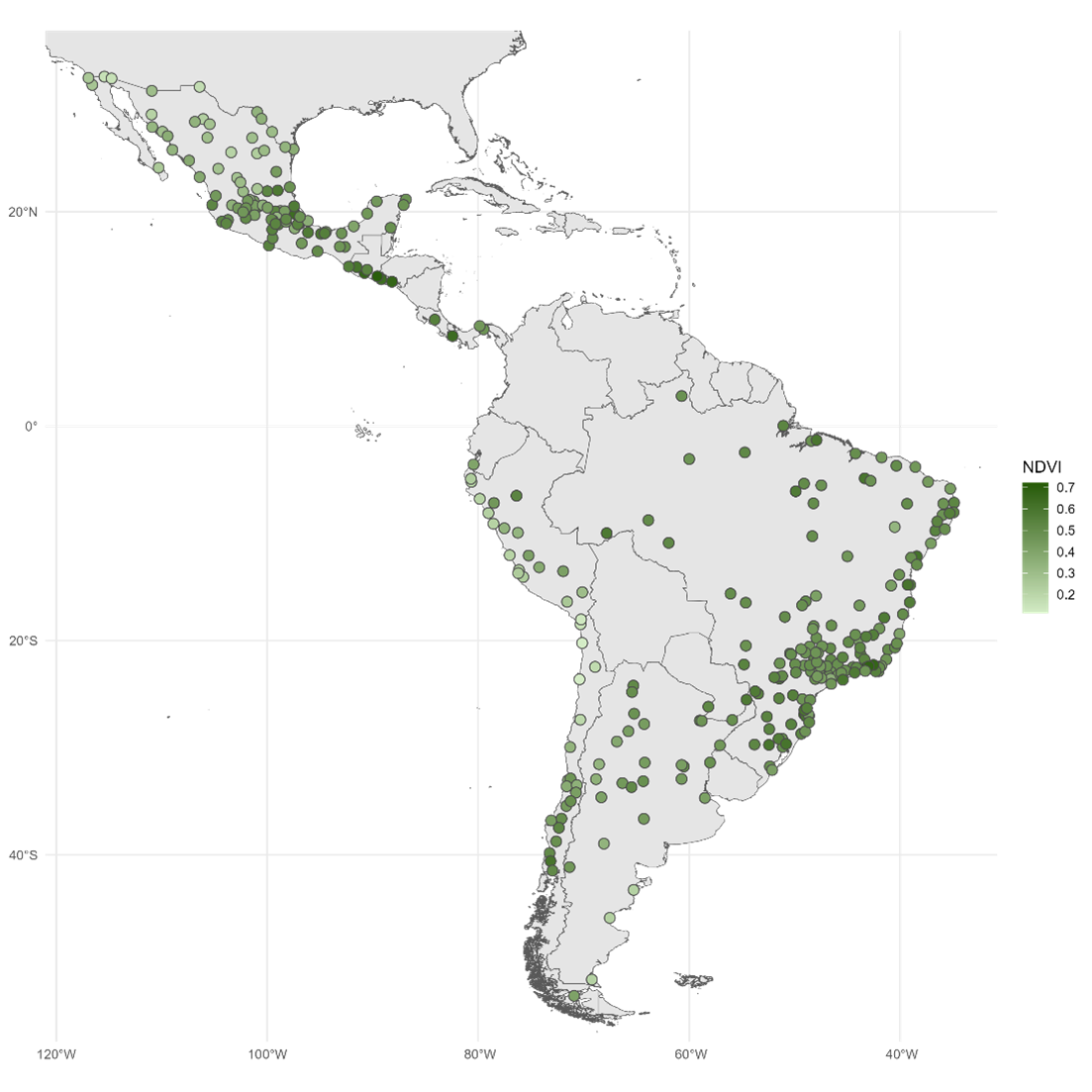
**

| **Appendix 9. Estimates of association between greenness, measured using population weighted normalized difference vegetation index (NDVI), and heat-relative risk (RR) estimates, stratified by climate zone.** | | | | | | | | |
| --- | --- | --- | --- | --- | --- | --- | --- | --- |
|  | **Arid climate zone cities**  **(N=79)** | | | | **Temperate and tropical climate zone cities**  **(N=244)** | | | |
| **Greenness (NDVI)** | **Beta** | **95% CI** | **AIC** | **Cochran’s Q (p - value)** | **Beta** | **95% CI** | **AIC** | **Cochran’s Q (p -value)** |
| **Low** | **REF** |  |  |  | **REF** |  |  |  |
| Moderate | -0.019 | -0.07, 0.04 | -65.0 | 83.7 (0.11) | -0.005 | -0.03, 0.02 | -282.3 | 314.9 (<0.01) |
| High | -0.009 | -0.08, 0.06 |  |  | -0.001 | -0.03, 0.03 |  |  |
| Abbreviations: AIC, Akaike Information criterion; CI, confidence interval; EDF, excess death fractions; NDVI, normalized difference vegetation index.  ^1^The reference category is cities in the lowest tertile of the climate-zone specific distribution of greenness, as measured by NDVI. Moderate and high levels of greenness refer to the second and third tertiles of the climate zone specific distribution of greenness, measured by the NDVI. The results were derived from random effects meta-regression models that were adjusted for particulate matter < 2.5 µg/m^3^, social environment index, country group, and a green space clustering metric. Models were run separately for arid and non-arid climate zone cities. | | | | | | | | |

| **Appendix 10. Estimates of association between greenness, measured using the normalized difference vegetation index (NDVI), and heat excess death fractions, stratified by climate zone.^1^ In these sensitivity analyses, models were adjusted for population density, in addition to particulate matter < 2.5 µg/m^3^, social environment index, country group, and clumpiness. Results are presented as % change in the heat excess death fraction associated with moving from low to moderate, and low to high levels of NDVI.** | | | | | | |
| --- | --- | --- | --- | --- | --- | --- |
|  | **Arid climate zone cities**  **(N=79)** | | | **Non-arid climate zone cities**  **(N=244)** | | |
| **Greenness (NDVI)** | **Beta** | **95% CI** | **AIC** | **Beta** | **95% CI** | **AIC** |
| **Low** | **REF** |  |  | **REF** |  | 784.5 |
| Moderate | -0.40 | -1.06, 0.26 | 288.7 | -0.04 | -0.25, 0.17 |  |
| High | -0.21 | -0.94, 0.52 |  | 0.05 | -0.19, 0.29 |  |
| Abbreviations: AIC, Akaike Information criterion; CI, confidence interval; EDF, excess death fractions; NDVI, normalized difference vegetation index.  ^1^The reference category is cities in the lowest tertile of the climate-zone specific distribution of greenness, as measured by NDVI. Moderate and high levels of greenness refer to the second and third tertiles of the climate zone specific distribution of greenness, measured by the NDVI. The results were derived from random effects meta-regressions that were adjusted for particulate matter < 2.5 µg/m^3^, social environment index, country group, population density, and clumpiness, which is a measure of green space clustering. Models were run separately for arid and non-arid climate zone cities. | | | | | | |

| **Appendix 11. Estimates of association between greenness, measured using the normalized difference vegetation index (NDVI), and heat excess death fractions, stratified by climate zone.^1^ In these sensitivity analyses, cities with excess death fractions < 0 were excluded.** | | | | | | |
| --- | --- | --- | --- | --- | --- | --- |
|  | **Arid climate zone cities**  **(N=66)** | | | **Non-arid climate zone cities**  **(N=177)** | | |
| **Greenness (NDVI)** | **Beta** | **95% CI** | **AIC** | **Beta** | **95% CI** | **AIC** |
| **Low** | **REF** |  |  | **REF** |  | 784.5 |
| Moderate | -0.34 | -1.17, 0.49 | 211.4 | 0.04 | -0.27, 0.35 |  |
| High | -0.31 | -1.37, 0.76 |  | -0.07 | -0.43, 0.29 |  |
| Abbreviations: AIC, Akaike Information criterion; CI, confidence interval; EDF, excess death fractions; NDVI, normalized difference vegetation index.  ^1^The reference category is cities in the lowest tertile of the climate-zone specific distribution of greenness, as measured by NDVI. Moderate and high levels of greenness refer to the second and third tertiles of the climate zone specific distribution of greenness, measured by the NDVI. The results were derived from random effects meta-regressions that were adjusted for particulate matter < 2.5 µg/m^3^, social environment index, country group, population density, and clumpiness, which is a measure of green space clustering. Models were run separately for arid and non-arid climate zone cities. | | | | | | |
